# The Time Course of Monocytes Infiltration After Acoustic Overstimulation

**DOI:** 10.3389/fncel.2022.844480

**Published:** 2022-04-12

**Authors:** Seung Ho Shin, Jinsei Jung, Haeng Ran Park, Nam Suk Sim, Jae Young Choi, Seong Hoon Bae

**Affiliations:** ^1^Department of Otorhinolaryngology-Head and Neck Surgery, Yongin Severance Hospital, Yonsei University College of Medicine, Yongin, South Korea; ^2^Department of Otorhinolaryngology, Yonsei University College of Medicine, Seoul, South Korea; ^3^Graduate School of Medical Science, Brain Korea 21 Project, Yonsei University College of Medicine, Seoul, South Korea

**Keywords:** acoustic overstimulation, noise-induced hearing loss, cochlea, macrophage, monocyte, neutrophil

## Abstract

Cochlea macrophages regulate cochlea inflammation and may harbors the potentials to protect hearing function from injury, including acoustic overstimulation. Cochlea macrophage numbers increase at 3–7 days after acoustic stimulation. However, the exact timing of macrophage infiltration and maturation from inflammatory monocytes is unclear. Furthermore, neutrophils may also be involved in this process. Therefore, in this study, we investigated time-dependent immune cell infiltration, macrophage transformation, and neutrophil involvement following acoustic stimulation. Flow cytometry and immunofluorescence were conducted in C-X3-C motif chemokine receptor 1 (CX3CR1)^+/GFP^ mice after acoustic overstimulation (at baseline and at 1, 2, 3, and 5 days after exposure to 120 dB for 1 h) to identify inflammatory monocytes in the cochlea. RNA-sequencing and quantitative polymerase chain reaction were performed to identify differentially expressed genes. Inflammatory monocytes infiltrated into the lower portion of the lateral wall within 2 days after acoustic overstimulation (dpn), followed by transformation into macrophages at 3–5 dpn *via* CX3CR1 upregulation and Ly6C downregulation. In addition, inflammatory monocytes were aggregated inside the collecting venule only at 1 dpn. Neutrophils were not a major type of phagocyte during this response. The gene encoding C-C motif chemokine ligand 2 gene was significantly upregulated as early as 3 h after acoustic overstimulation. Given these results, treatment to control immune response after a noise-induced hearing loss should be applied as soon as possible.

## Introduction

Acoustic overstimulation results in marked cochlea changes related to hearing function, structural properties, and immunological reactions (Suzuki et al., 2002; Wang et al., 2011; Tan et al., 2016; Kobel et al., 2017). Mainly the innate immune system is involved in inflammation of the cochlea after acoustic overstimulation (Frye et al., 2019; He et al., 2020). Hirose et al. first demonstrated the presence of CD45-positive cells in the cochlear parenchyma, and these leukocytes have subsequently been shown to have characteristics consistent with macrophages (Hirose et al., 2005; Okano et al., 2008; Shi, 2010). Inflammation induced by acoustic overstimulation increases the population of macrophages in the cochlea, and bone marrow-derived monocytes from peripheral blood stream are thought to transform into macrophages to facilitate the clearance of degenerated cell debris (Hirose et al., 2005; Shi, 2010; He et al., 2020). However, few studies have provided direct evidence that the increased population of macrophages originates mainly from monocytes in the peripheral blood stream. Furthermore, the temporal and spectral properties of infiltrated monocytes have not been fully elucidated.

It is a well-known cochlear innate immune response after acoustic overstimulation that macrophages are increased in the cochlea from 3 to 7 days postnoise (dpn) (Hirose et al., 2005; Tornabene et al., 2006; Yang et al., 2015; He et al., 2020). Neutrophils, the first-line phagocytes involved in the typical inflammatory response, have not been extensively studied in the context of acoustic overstimulation-induced inflammation (Fujioka et al., 2014; Hu et al., 2018). However, several recent studies have demonstrated increased neutrophil numbers in the cochlea at 1 dpn (Rai et al., 2020; Zhang et al., 2021). Unlike their known roles in lipopolysaccharide-induced or infectious inflammation, the involvement of neutrophil infiltration in acoustic overstimulation-induced cochlear inflammation is still unclear (Bae et al., 2021b).

Resident macrophages in the cochlea express high levels of the fractalkine receptor C-X3-C motif chemokine receptor 1 (CX3CR1) (Kaur et al., 2015a,b; Hirose et al., 2017; Bae et al., 2021a). Inflammatory monocytes in the peripheral blood show a CX3CR1^low^/Ly6C^high^ profile (Hamon et al., 2017; Meghraoui-Kheddar et al., 2020). Therefore, in this study, we investigated the transformation of immunological markers during cochlear inflammation, with a focus on inflammatory monocytes destined to transform into macrophages. We also evaluated the time course of inflammatory monocyte infiltration and transformation using a quantitative approach. Finally, we characterized neutrophil infiltration in the context of acoustic overstimulation-induced cochlear inflammation. Our findings provide important insights into the regulation of innate immune responses in the cochlea.

## Materials and Methods

### Animals

Wild-type C57BL/6 mice and CX3CR1^GFP/GFP^ transgenic C57BL/6 mice were purchased from Jackson Laboratory (ME, United States) *via* Orient Bio (Sungnam, Republic of Korea). CX3CR1^GFP/+^ mice were used to visualize monocytes and macrophages. The mice were housed and maintained according to the requirements for animal research established by Yonsei University Health System, and all procedures were approved by the Institutional Animal Care and Use Committee (approval number 2019-0182). Mice (4–12 weeks old, both sexes) were fed *ad libitum* and housed in cages in an environmentally controlled room under a 12-h light cycle. We used 4 mice for validating permanent hearing loss after acoustic overstimulation, 8 mice in each condition for flow cytometry experiment, 3 mice in each condition for imaging studies, 4 mice in each condition for polymerase chain reactions, and 3 mice in each group for RNA sequencing.

### Noise Generation and Audiologic Evaluation

White noise (300–10,000 Hz) was generated using a personal computer and an amplifier (R-399; Inter M, Seoul, South Korea) and delivered through speakers (290-8L; Altec Lansing, Oklahoma City, OK, United States) in a noise booth. Mice were continuously exposed to a 120-dB peak equivalent sound pressure level for 1 h to produce permanent threshold shift and acoustic overstimulation-induced cochlear inflammation.

The auditory brainstem response (ABR) was measured after anesthetizing the mice with xylazine [20 mg/kg, intraperitoneal injection (IP)] and ketamine hydrochloride (30 mg/kg IP). The hearing level of each mouse was checked by measuring the ABR threshold with a TDT auditory evoked potential workstation (Tucker-Davis Technologies, Alachua, FL, United States). Both ears of each mouse were stimulated with an ear probe sealed in the ear canal. The ABRs to click and tone burst stimuli were recorded, and thresholds were obtained for each ear. The ABRs were measured before and at 2 and 7 dpn.

### Histological Preparation and Immunostaining

After sacrificing the mice in a CO_2_ chamber, the bilateral temporal bones were dissected and fixed in 4% paraformaldehyde for 24 h at 4°C after local perfusion with a fixative through the oval and round windows. After fixation, the samples were incubated in 0.125M ethylenediaminetetraacetic acid (ETDA) solution for 24 h at 4°C for decalcification. To obtain lateral wall samples, decalcified cochleae were cut in half through the apex-oval window axis. Under optical microscopy, the cochlear lateral wall of the basal turn was carefully separated using forceps and microscissors. Other structures, including the organ of Corti, Reissner’s membrane, and modiolus, were trimmed.

The tissues were blocked with 10% donkey serum and incubated with allophycocyanin (APC)-conjugated anti-Ly6C antibodies (HK1.4; cat. no. 128016; Biolegend) at 4°C overnight. The samples were then mounted with a mounting solution (Sigma-Aldrich, St. Louis, MO, United States) and viewed under an LSM980 confocal microscope (Zeiss, Jena, Germany). All immunostaining experiments were repeated in three biological replicates per time point. For three-dimensional (3D) imaging, lateral wall samples were incubated with fluorescein isothiocyanate-conjugated anti-Ly6C antibodies (HK1.4; cat. no. 128005; Biolegend) at 4°C overnight. The samples were then directly investigated under a two-photon microscope (LSM7MP; Carl-Zeiss, Germany) using the same method as in our previous study (Bae et al., 2021b).

We calculated the outer hair cell count as described previously (Bae et al., 2020). Briefly, after fixation and decalcification as described above, the cochlea was separated into apical and basal portions under an optical microscope. Then, the organ of Corti was directly visualized under a two-photon microscope (LSM7MP; Carl-Zeiss).

### Intravital Imaging

We performed intravital imaging of the collecting venule as described previously (Bae et al., 2021b). Briefly, the cochlea of anesthetized CX3CR1^GFP/+^ mice was exposed by surgery, and Texas-red conjugated dextran (500 μg/animal) was injected into the retro-orbital sinus. The stapedial artery was ligated, and the bony capsule of the basal turn was carefully drilled until the collecting venule could be visualized by optical microscopy. Then, a two-photon microscope (LSM7MP; Carl-Zeiss) was used to acquire imaging data. Images were obtained for 30 min (1 frame/min). The cells that appeared in four consecutive sequences (a 3-min interval) were selected manually and connected to create a track using IMARIS software (Bitplane AG, Zurich, Switzerland).

### Flow Cytometry

Cochleae were obtained from mice following cardiac perfusion with phosphate-buffered saline (PBS). We carefully removed the soft tissues from the cochlea in ice-cold PBS. The cochlea was then transferred to a new dish filled with ice-cold PBS, and the bony capsule was carefully removed, ensuring that the bone marrow was not exposed. We discarded the remnants after extracting the inner tissue of the cochlea, including the lateral wall, the organ of Corti, and modiolus, through the opening. The tissue from the cochlea was collected and trypsinized (0.25% trypsin/ETDA solution) for 10 min at 37°C. The trypsinized tissue was ground on a filter (pore size, 40 μm). Peripheral blood was obtained by cardiac puncture before cardiac perfusion. Samples were treated with red blood cell lysis buffer to remove red blood cells, and the remaining cells were stained with antibodies at a 1:200 dilution for 30 min. Samples were then analyzed using a FACSverse II BD flow cytometer (BD Biosciences, Sparks, MD, United States). One sample consisted of four cochleae from two mice, and four samples were included in each group. The flow cytometry data were managed using FlowJo software (Tree Star, Ashland, OR, United States). Peripheral blood was used as a reference for gating strategies for inflammatory monocytes in the cochlea because the immune cells infiltrated from the peripheral blood (Supplementary Figures 1, 2).

7-Aminoactinomycin D (7-AAD; cat. no. 420403; Biolegend) was used for live cell gating, phycoerythrin (PE)-Cy7-conjugated anti-CD11b (M1/70; cat. no. 25-0112-82; Invitrogen, Carlsbad, CA, United States) was used for myeloid cell gating, PE-conjugated anti-Ly6G (1A8; cat. no. 127608; Biolegend) was used for neutrophil gating, APC-conjugated anti-F4/80 (BM8; cat. no. 17-4801-82; Invitrogen) was used for monocyte/macrophage gating, and APC-Cy7-conjugated anti-Ly6C (HK1.4; cat. no. 128026; Biolegend) was used for inflammatory monocyte gating (Supplementary Figures 1, 2). For monocyte depletion, 0.2 mL (5 mg/mL) clodronate liposomes (Liposoma) was intraperitoneally injected into mice 1 day before acoustic overstimulation.

### RNA-Sequencing Analysis

Bilateral temporal bones were separated from the body immediately after the sacrifice. Immediately after the cochlea was isolated from the vestibule, it was immersed in TRIzol reagent (Invitrogen). Three biological replicates (6 cochleae from 3 mice) were used in each group, untreated and 3 h after noise exposure. Transcriptome sequencing was performed using an Illumina platform. We used the HISAT2 software to map the reads to the mouse genome (mm10_NCBI_108) and generate gene expression values in the normalized form of transcripts per kilobase million values. All differentially expressed genes (DEGs) were selected based on the following cut offs: *p* < 0.05 and |fold change| ≥ 2. Gene-set enrichment analysis and pathway analysis were conducted using public databases. Functional enrichment with Gene Ontology (GO) was performed using the g:Profiler.^1^ The Kyoto Encyclopedia of Genes and Genomes (KEGG) database was used to analyze pathway enrichment.^2^

### Reverse Transcription Quantitative Real-Time Polymerase Chain Reaction

Bilateral temporal bones were separated from the body immediately after the sacrifice. Immediately after the cochlea was isolated from the vestibule, it was immersed in TRIzol reagent (Invitrogen). Four biological replicates (8 cochleae from 4 mice) were used in each group that was 3 h, 1 day, 5 days after noise exposure, and untreated control. Total RNA was isolated and purified using a PureLink RNA mini kit (Ambion, Austin, TX, United States), according to the manufacturer’s instructions. RNA samples from the cochlea were reverse-transcribed using an iScript Select cDNA Synthesis Kit (Bioneer, South Korea). We performed RT-qPCR using Taqman Master Mix (Applied Biosystems, Foster City, CA, United States). The PCR mixture contained 400 μg cDNA and primers. To quantify the mRNA levels of interleukin 1 beta (*Il1-*β), interleukin-6 (*Il-6*), and C-C motif chemokine ligand 2 (*Ccl2*) in the cochlea, we used gene-specific primer pairs with glyceraldehyde 3-phosphate dehydrogenase (*GAPDH*) control primers for normalization and TaqMan probes (Applied Biosystems). Amplification and quantification were performed using an ABI 7500 RT-PCR system (Applied Biosystems). Assay ID of used TaqMan probes are listed below.

*Il1-*β: Mm00434228_m1

*Il-6*: Mm00446190_m1

*Ccl2*: Mm00441242_m1

*GAPDH*: Mm99999915_g1

### Statistical Analyses

To compare multiple groups, we performed one-way analysis of variance (ANOVA) and *post hoc* Tukey’s multiple comparisons test. To compare multiple time points in multiple groups, we performed two-way ANOVA and *post hoc* Dunnet’s multiple comparisons test. All data are presented as means and standard errors of the means (error bars). SPSS 25.0 (IBM, Armonk, NY, United States) and Prism 8.0 (GraphPad Software, San Diego, CA, United States) were used for statistical analyses. Results with *P* values less than 0.05 were considered statistically significant.

## Results

### Inflammatory Monocytes From Peripheral Blood Infiltrated Into the Cochlea After Acoustic Overstimulation

**FIGURE 1:**
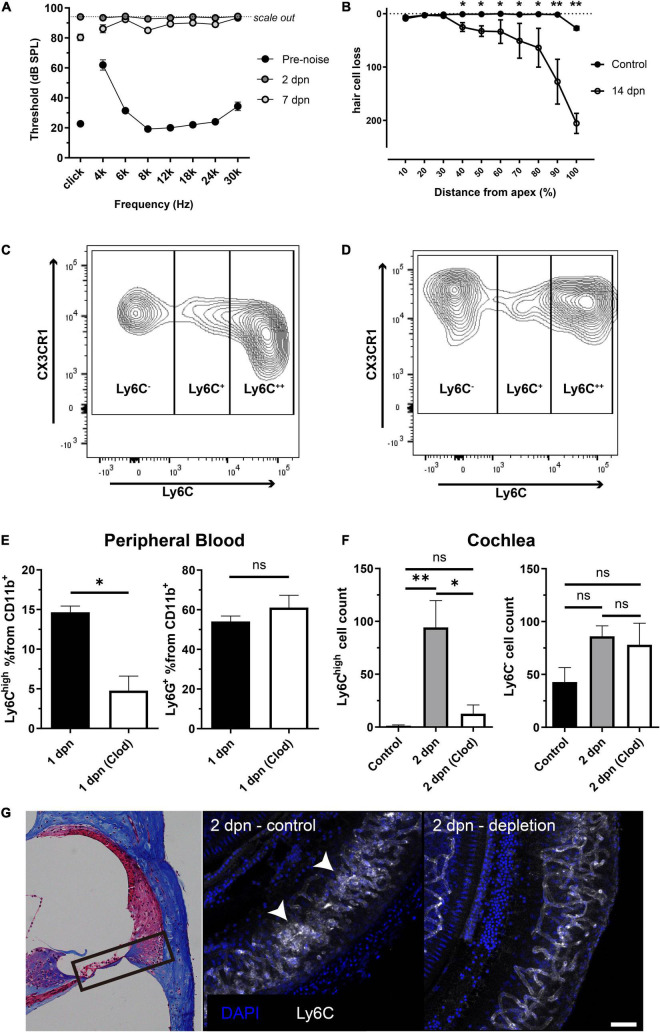
Inflammatory monocytes from the bloodstream infiltrated the cochlea after acoustic overstimulation. **(A)** Effects of acoustic overstimulation (120 dB, 1 h white band noise exposure) on the permanent threshold shift (*N* = 8 ears). **(B)** Effects of noise exposure on outer hair cell degeneration (*N* = 4 ears in each group). **(C)** Effects of acoustic stimulation on peripheral blood CD11b^+^Ly6G^–^F4/80^+^CX3CR1^+^ populations and distinction between Ly6C^++^ inflammatory monocytes and Ly6C^–^ patrolling monocytes. **(D)** Effects of acoustic overstimulation on cell populations (CD11b^+^Ly6G^–^F4/80^+^CX3CR1^+^ cells; Ly6C^++^ possible inflammatory monocytes and Ly6C^–^ possible resident macrophages) in cochlea at 2 days. **(E)** Clodronate liposome successfully reduced monocytes in peripheral blood (left) compared to neutrophils (right) in 1 dpn at which the monocytes begin to infiltrate to the cochlea. **(F)** Effects of clodronate liposome treatment after acoustic overstimulation on Ly6C^++^ possible inflammatory monocyte populations in the cochlea after 2 days. (left) Effects of clodronate liposome treatment after acoustic overstimulation on Ly6C^–^ possible resident macrophage populations. (right) *Clod*: clodronate liposome-treated group. **: *p* < 0.01, *: *p* < 0.05, *ns*: not significant. **(G)** Immunofluorescence study comparing control and clodronate liposome at 2 dpn. White arrows indicate clusters of monocytes. The black box in the light microscopy image located on the left indicates the anatomical location of the immunofluorescence images. Scale bar = 50 μm.

Acoustic overstimulation resulted in a permanent threshold shift in the hearing function of mice at all examined frequencies (Figure 1A). Importantly, this hearing function did not recover after 2 or 7 dpn. Outer hair cell degeneration was also identified in the noise-exposed cochlea, particularly in the basal turn, which is known to be vulnerable to acoustic overstimulation (Sha et al., 2001) (Figure 1B). In previous studies, increased macrophage infiltration was observed from 3 dpn, and blood-derived monocytes seem to transform into macrophages after infiltration (Hirose et al., 2005; Shi, 2010; He et al., 2020). Therefore, in this study, we investigated the infiltration of monocytes into the cochlea at 2 dpn. Indeed, flow cytometry analysis indicated that the 2 dpn cochlea contained CD11b^+^Ly6G^–^F4/80^+^Cx3cr1^+^Ly6C^++^ cells, which are inflammatory monocytes based on flow cytometry data from the peripheral blood (Figures 1C,D and Supplementary Figure 1).

Next, we treated peripheral blood with clodronate liposomes to deplete monocytes and confirm that the monocytes in the 2 dpn cochlea were recruited from the blood stream. Clodronate liposomes significantly reduced the infiltration of monocytes into the 2 dpn cochlea. The absolute cell count of the CD11b^+^Ly6G^–^F4/80^+^CX3CR1^+^Ly6C^–^ population, representing resident macrophages, was not significantly different between groups (Figures 1E–G). Taken together, these findings suggested that the inflammatory monocytes infiltrating into the cochlea after acoustic overstimulation were mainly derived from the peripheral blood.

### Monocytes Infiltrated Into the Cochlea Within 2 Dpn, With Limited Neutrophils

**FIGURE 2:**
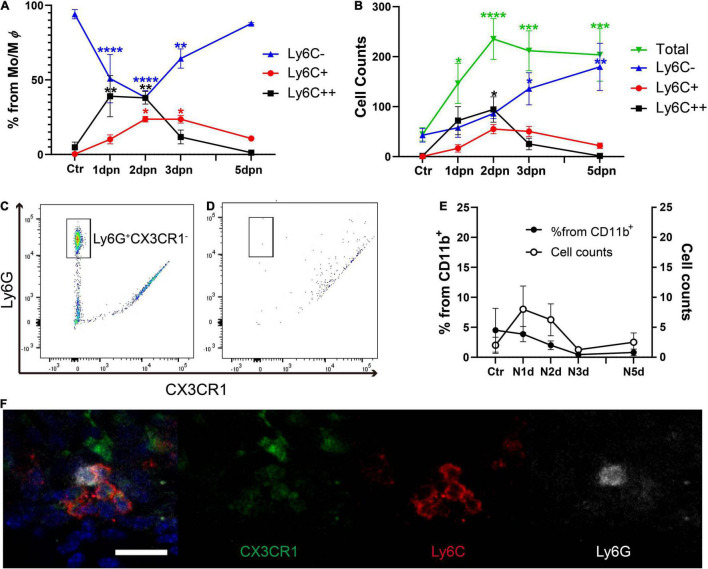
Inflammatory monocytes infiltrated within 2 days after acoustic stimulation, whereas neutrophil infiltration was not observed. **(A)** Percentages of cells sorted by Ly6C expression from the CD11b^+^Ly6G^–^F4/80^+^CX3CR1^+^ population, demonstrating Ly6C^++^ cell infiltration within 2 days after acoustic overstimulation. **(B)** Total cell counts showing the CD11b^+^Ly6G^–^F4/80^+^CX3CR1^+^ population did not change after 2 dpn. **(C)** Distinction between Ly6G^+^CX3CR1^–^ neutrophils and CD11b^+^ cells in the peripheral blood. **(D)** Lack of distinct neutrophils in the cochlea at 1 day after acoustic overstimulation compared with CD11b^+^ cells. **(E)** Changes in the percentages and the cell count of neutrophils in cochlea after acoustic overstimulation. There was no statistically significant value. *Ctr*: control, *N1d*: 1 day after noise, *N2d*: 2 days after noise, *N3d*: 3 days after noise, *N5d*: 5 days after noise, *Mo*: monocytes, *M*φ: macrophages. ****: *p* < 0.0001, ***: *p* < 0.001, **: *p* < 0.01, *: *p* < 0.05, when compared with the control. **(F)** Immunofluorescence study of lower portion of spiral ligament at 1 dpn. Neutrophil (white) is mixed in the cluster of inflammatory monocytes (red and weak green). Scale bar = 20 μm.

Next, we conducted serial flow cytometry analysis to determine the time course of the monocyte/macrophage response in the cochlea after acoustic overstimulation. Inflammatory monocytes were significantly increased at 1 and 2 dpn, followed by increases in macrophage numbers (Figures 2A,B). Interestingly, the total CD11b^+^Ly6G^–^F4/80^+^CX3CR1^+^ population was not significantly changed after 2 dpn, suggesting that the infiltration of monocytes was limited within 2 dpn and that these cells may subsequently be transformed into macrophages.

Next, we evaluated CD11b^+^Ly6G^+^CX3CR1^–^ neutrophils using different gating strategies in the same dataset (Figures 2C,D and Supplementary Figure 2). For clear distinguishment of neutrophils, CD11b + myeloid cells were sorted by GFP and PE signals to exclude the possible spillover phenomenon resulting from PE-conjugated anti-Ly6G antibodies and green fluorescent protein (GFP)-CX3CR1 reporter mice. The Ly6G^+^CX3CR1^–^ neutrophils were distinct in the peripheral blood. Interestingly, neutrophils were rare (less than 5% in average from the CD11b^+^ population) at any time point after acoustic overstimulation (Figure 2E). However, although statistically insignificant, several neutrophils were detected in 1 and 2 dpn. We next performed an immunofluorescence study of the lower spiral ligament, using Ly6C and Ly6G antibodies to confirm the presence of neutrophils. Indeed, several neutrophils were identified in the cluster of inflammatory monocytes at 1 dpn (Figure 2F). However, as the flow cytometry analysis indicated, the inflammatory monocytes were a substantially large number compared to neutrophils.

### Transformation Into Macrophages Occurred Within 5 Dpn

**FIGURE 3:**
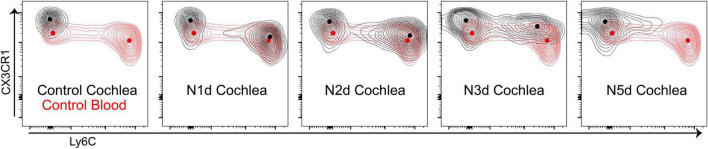
Infiltrated monocytes transformed into macrophages within 5 days after acoustic stimulation. Concatenated contour plots (merging four biological replicates for each time point) showing CD11b^+^Ly6G^–^F4/80^+^CX3CR1^+^ cells at different time points after acoustic overstimulation. *Black contour*: cochlea sample, *red contour*: control peripheral blood sample, *dots*: center of each population.

The density plot of the flow cytometry data was analyzed to investigate changes in the expression of Ly6C and CX3CR1 proteins (Figure 3). In the peripheral blood, there are two major CD11b^+^Ly6G^–^F4/80^+^CX3CR1^+^ populations showing differential expression of Ly6C; these populations are patrolling monocytes (Ly6C^–^) and inflammatory monocytes (Ly6C^++^) (Geissmann et al., 2003). When compared with patrolling monocytes in the peripheral blood, cochlear macrophages showed high expression of CX3CR1 at all time points. There was no newly observed population suspected to be infiltrated patrolling monocytes after acoustic overstimulation. By contrast, inflammatory monocytes abruptly appeared at 1 dpn. This population showed a gradual reduction in Ly6C expression and increase in CX3CR1 expression. Finally, these populations merged with the resident macrophage population at 5 dpn. We confirmed that there was no additional infiltration of inflammatory monocytes after 2 dpn; instead, the infiltrated monocytes were transformed into macrophages by 5 dpn.

### Monocytes Infiltrated the Lower Portion of the Spiral Ligament

**FIGURE 4:**
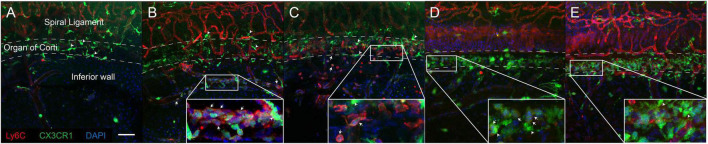
Inflammatory monocytes mainly infiltrated the lower portion of the lateral wall of the cochlea. Immunofluorescence studies of the lateral wall of the cochlea revealed that inflammatory monocytes (positive for Ly6C, weak CX3CR1) aggregated and infiltrated into the lower portion of the lateral wall after acoustic stimulation. **(A)** Control. **(B)** 1 dpn. **(C)** 2 dpn. These cells transform to macrophages expressing CX3CR1 with losing Ly6C after 2 dpn. **(D)** 3 dpn. **(E)** 5 dpn. Arrows indicate representative inflammatory monocytes (expressing both Ly6C and CX3CR1). Arrowheads indicate representative resident cochlea macrophages (expressing CX3CR1 only). *Red*: Ly6C, *green*: CX3CR1, *blue*: DAPI. Scale bar = 50 μm. Note that Ly6C also stained the capillary vessel wall.

According to the flow cytometry results, we performed immunofluorescence analysis of the lateral wall of the cochlea at 1 and 2 dpn (Figure 4). We observed distinct adherence of inflammatory monocytes in the collecting venule, which was located beneath the organ of Corti in the lateral wall, at 1 dpn (Figure 4B). Furthermore, in the cochlea at 2 dpn, the number of adhered cells appeared to decrease, and inflammatory monocytes were distributed in the lower part of the spiral ligament (Figure 4C). At 3 dpn and 5 dpn, CX3CR1 positive cells were increased and Ly6C signal of these cells was decreased compared to 1 dpn and 2 dpn (Figures 4D,E). These findings were consistent with the flow cytometry results and with the two-photon image using wild-type mice (Supplementary Figure 3). The crawling monocytes were also found in intravital imaging of the collecting venule in the basal turn at 1 dpn (Supplementary Video 1). Taken together, these findings suggested that the interaction between immune cells and the endothelium began at approximately 1 dpn and that infiltration mainly occurred at around 2 dpn.

### Genes Associated With Monocyte Infiltration Were Highly Expressed as an Early Response to Acoustic Overstimulation

**FIGURE 5:**
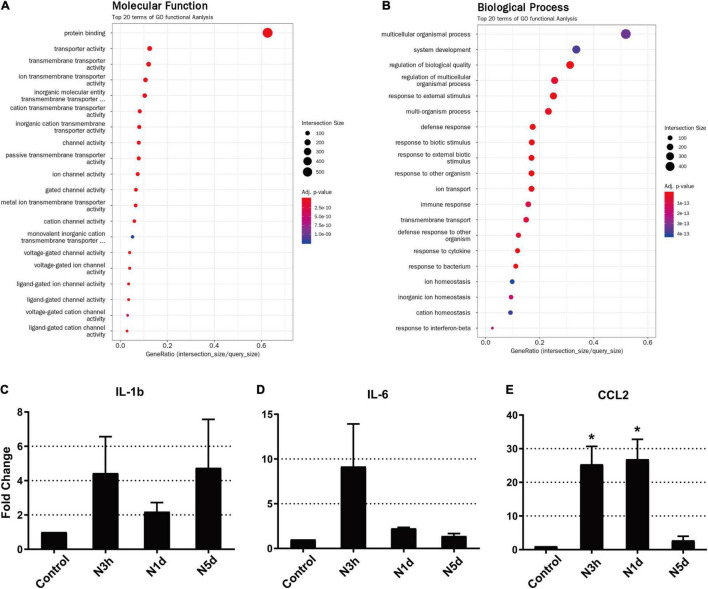
*Ccl2* was significantly upregulated as early as 3 h after acoustic overstimulation. **(A)** Gene Ontology analysis of molecular functions using RNA-sequencing data. **(B)** Gene Ontology analysis of biological processes. The top 20 terms are shown. **(C–E)** Expression of inflammatory molecules was analyzed by quantitative polymerase chain reaction (*N* = 4 biological replicates in each sample). *IL-1b*: interleukin 1 beta, *IL-6*: interleukin 6, *CCL2*: C-C motif chemokine ligand 2, *N3h*: 3 h after noise, *N1d*: 1 day after noise, *N5d*: 5 days after noise. *: *p* < 0.05.

Our results revealed that the infiltration of innate immune cells may occur earlier than expected. To support our results at the molecular level, we analyzed DEGs from RNA sequencing data to evaluate the expression of genes associated with monocyte infiltration at an extremely early stage (3 h) after acoustic overstimulation. In total, 585 genes were significantly different between control and noise-exposed cochleae. Among these genes, 340 were upregulated, and 245 were downregulated in noise-exposed cochleae compared with control cochleae. The GO analysis of molecular functions showed strong enrichment in protein binding function (Figure 5A). However, in the biological process category, processes associated with leukocyte migration were not within the top 20 terms (Figure 5B). In KEGG pathway analysis, the cytokine-cytokine receptor interaction pathway, which includes chemokines that attract monocytes from the blood flow, was the third most differentially expressed pathway (Table 1). Additionally, among the top 20 DEGs, four genes were chemokines (*Ccl2*, *Ccl7*, *Cxcl10*, and *Ccl12*; Table 2). The expression of *Ccl2* was increased by 29.89-fold. Importantly, qPCR results confirmed the RNA sequencing data, demonstrating significant increases in *Ccl2* expression at 3 h and 1 dpn (∼25 fold) and then a decrease to the baseline level at 5 dpn. Furthermore, *Il-1b* and *Il-6* expression levels increased shortly after acoustic stimulation, although these changes were not statistically significant (Figures 5C–E).

**TABLE 1:** Top 20 upregulated pathways in the KEGG database, sorted according to *p* value.

Pathway	Gene count	*p* value	FDR
Neuroactive ligand-receptor interaction	44	5.9E-22	1.7E-19
Metabolic pathways	81	2.2E-17	3.0E-15
Cytokine-cytokine receptor interaction	35	8.8E-17	8.3E-15
NOD-like receptor signaling pathway	30	2.2E-16	1.6E-14
TNF signaling pathway	23	19.1E-15	1.1E-13
Glutamatergic synapse	22	1.9E-14	8.8E-13
Influenza A	25	3.6E-14	1.5E-12
Calcium signaling pathway	26	9.0E-14	3.2E-12
Hepatitis C	23	1.1E-12	3.3E-11
Circadian entrainment	19	1.7E-12	4.7E-11
Oxytocin signaling pathway	22	3.6E-12	9.1E-11
IL-17 signaling pathway	18	5.5E-12	1.3E-10
Measles	21	9.7E-12	2.1E-10
Osteoclast differentiation	20	1.1E-11	2.3E-10
African trypanosomiasis	13	4.2E-11	8.0E-10
Nicotine addiction	13	7.0E-11	1.2E-9
MAPK signaling pathway	27	8.7E-11	1.4E-9
Epstein-Barr virus infection	24	1.1E-10	1.7E-9
Viral protein interaction with cytokine	17	2.7E-10	4.0E-9
Retrograde endocannabinoid signaling	19	7.4E-10	1.0E-8

**TABLE 2:** Top 20 upregulated genes, sorted according to *p* value.

Symbol	Gene name	Fold change	*p* value
*Ccl2*	Chemokine (C-C motif) ligand 2	29.89	4.1E-37
*Fosl1*	Fos-like antigen 1	151.49	1.0E-29
*Ccl7*	Chemokine (C-C motif) ligand 7	17.57	9.7E-28
*Osmr*	Oncostatin M receptor	5.58	1.4E-26
*Zbp1*	Z-DNA binding protein 1	8.61	1.6E-25
*Cxcl10*	Chemokine (C-X-C motif) ligand 10	24.55	3.3E-25
*Tnfrsf12a*	Tumor necrosis factor receptor superfamily, member 12a	5.87	6.1E-25
*Socs3*	Suppressor of cytokine signaling 3	6.04	1.2E-23
*Ccl12*	Chemokine (C-C motif) ligand 12	7.61	2.0E-22
*Ifi27l2b*	Interferon, alpha-inducible protein 27 like 2B	1240.83	9.4E-22
*Ier3*	Immediate early response 3	3.77	1.3E-20
*Maff*	V-maf musculoaponeurotic fibrosarcoma oncogene family, protein F (avian)	6.33	3.3E-20
*Hmga1-rs1*	High mobility group AT-hook I, related sequence 1	4.76	4.7E-19
*Atf3*	Activating transcription factor 3	13.63	7.3E-19
*Tgfbi*	Transforming growth factor, beta induced	2.98	5.9E-18
*Krt8*	Keratin 8	6.54	9.5E-17
*Timp1*	Tissue inhibitor of metalloproteinase 1	9.68	1.4E-16
*Gdf15*	Growth differentiation factor 15	15.47	2.3E-16
*Isg15*	ISG15 ubiquitin-like modifier	6.61	3.7E-16
*Gem*	GTP binding protein (gene overexpressed in skeletal muscle)	4.72	4.3E-16

## Discussion

In this study, we demonstrated that inflammatory monocytes infiltrated exclusively between 1 and 2 dpn, after which the infiltrated monocytes transformed into macrophages by upregulating CX3CR1 and downregulating Ly6C. Our findings showed that immune response associated with immune cell infiltration began earlier than previously expected and that the infiltration process was brief. In the lateral wall, the infiltration process occurred mainly in the lower portion of the spiral ligament and collecting venule. In addition, an influx of massive neutrophils was not observed after acoustic overstimulation.

Numerous studies have reported increases in cochlear macrophage populations after acoustic overstimulation; however, few reports have focused on monocytes as precursors of macrophages in the bloodstream. Because the regulation of macrophages is believed to have the potential to alleviate cochlear damage induced by noxious stimulation, including acoustic overstimulation, aging, and ototoxic drugs, researchers have focused on the functions and responses of macrophages under such condition (Sato et al., 2010; Kaur et al., 2015b; Frye et al., 2017; Wood and Zuo, 2017; Noble et al., 2021). In our previous study, we described inflammatory monocyte infiltration at 1 dpn, and we speculated that this monocyte infiltration process may be brief, e.g., within several days after noise exposure (Bae et al., 2021c). Indeed, in the current study, we found that the infiltration process was complete within 2 dpn, and monocytes then transformed into macrophages. Accordingly, the increased macrophage population at 3–7 dpn described in previous studies may be explained by the transformation of macrophages. In addition, we confirmed that these increased macrophages originated mostly from blood-derived monocytes. Clodronate liposomes, which are widely used to deplete monocytes/macrophages, also significantly reduced the numbers of infiltrated monocytes in the cochlea at 2 dpn (Summan et al., 2006; Kreisel et al., 2010; Waltl et al., 2018). By contrast, the population of resident macrophages was not altered after clodronate liposome treatment. The preservation of cochlear resident macrophages from clodronate is maybe because of the blood-labyrinthine barrier which is consisted of tight junctions that prevent clodronate diffuse into the cochlear parenchyma. The collecting venule, in which immune cells aggregated at 1 dpn, seemed to be the main gateway for cell infiltration in the lateral wall. In general, immune cells infiltrate through the venular walls because the blood flow is slower and because it is easier to breach the vessel wall than the arteriole (Voisin and Nourshargh, 2013). Notably, the cochleae also have collecting venules in the lower part of the spiral ligament and modiolus. Previous studies have reported upregulation of intercellular adhesion molecule-1 (ICAM-1), a key endothelial protein involved in immune cell trafficking in the cochlea after acoustic overstimulation (Harris et al., 1990; Suzuki and Harris, 1995). Consistent with our findings, Tan et al. showed that upregulation of *Icam-1* after acoustic overstimulation peaks at 1 dpn in the lower part of the spiral ligament and that *Ccl2* is also significantly upregulated 6 h after acoustic overstimulation (Tan et al., 2016). Taken together, these findings supported that the lower part of the spiral ligament was the main gateway for immune cell infiltration in the cochlear lateral wall.

The early response of the cochlea after acoustic overstimulation was supported by RNA sequencing and qPCR. In particular, the *Ccl2* gene, which is the most important cytokine mediating the recruitment of monocytes/macrophages, memory T lymphocytes, and natural killer cells, was substantially upregulated as early as 3 h after acoustic overstimulation (Gu et al., 1999). Furthermore, the early expression of genes associated with immune cell infiltration, including *Ccl2*, has also been reported in previous studies investigating the cochlea at 2, 3, and 12 h after acoustic overstimulation (Maeda et al., 2018, 2021). The response of resident macrophages to acoustic overstimulation seems to be immediate and strong. However, the response is limited to monocytes and does not largely affect neutrophils. The reason for this exclusion of neutrophils from acoustic overstimulation-induced inflammation is unclear. A recent study shed light on this issue that CX3CR1 has a role in inhibiting neutrophil infiltration after acoustic overstimulation because CX3CR1 null mice showed neutrophil infiltration at 1 dpn (Zhang et al., 2021). In addition, the result of the study by Zhang et al. is consistent with our data that a limited number of neutrophils were identified in CX3CR1^GFP/+^ mice after acoustic overstimulation. Another interesting study by the same group was published, they used monocyte tracking using latex beads. The results were consistent with ours that monocytes were found in the cochlea at 1 dpn (Zhang et al., 2020). The discrepancy between our results and study demonstrating neutrophil infiltration at 1 day after acoustic overstimulation may be related to the anti-Ly6G antibody used in flow cytometry analysis or immunofluorescence (Rai et al., 2020). The anti-Ly6G antibody was manufactured mainly from two clones (RB6-8C5 and 1A8). The Ly6G clone RB6-8C5 (also called an anti-Gr-1 antibody), which was used in the study showing neutrophil infiltration after acoustic overstimulation, has been reported to bind to Ly6C (Fleming et al., 1993; Rose et al., 2012; Lee et al., 2013). Therefore, cells showing positivity in staining with RB6-8C5 may include inflammatory monocytes that strongly express Ly6C. Our results showed that CD11b^+^Ly6G^+^CX3CR1^–^ neutrophils were distinctly present in the peripheral blood but there was no massive infiltration of these cells in the cochlea after acoustic overstimulation. Ly6C-positive cells, which were aggregated on the collecting venule at 1 dpn also showed a weak green signal (representing CX3CR1) in immunofluorescence analyses. Moreover, the fact that neutrophils were not affected by clodronate liposome also supported that the Ly6C-positive cell population in our study was not neutrophils (Van Rooijen and Sanders, 1994). Consequently, we concluded that the CD11b^+^Ly6G^–^F4/80^+^CX3CR1^+^Ly6C^++^ cell population, which abruptly increased 1–2 days after acoustic overstimulation, consisted of inflammatory monocytes, rather than neutrophils.

The function of the CX3CR1 protein is still unclear. *Cx3cr1*-null mice are vulnerable to ototoxic drugs and acoustic overstimulation (Sato et al., 2010; Kaur et al., 2015b,^2019^; Zhang et al., 2021). As a C-X 3 C motif chemokine, CX3CR1 is likely to modulate the inflammatory response and may be involved in immune cell infiltration (Bazan et al., 1997; Stievano et al., 2004). In the cochlea, deficiency of CX3CR1 reduces the infiltration of macrophages during inflammation, resulting in increased severity of damage (Sato et al., 2010; Kaur et al., 2015b,^2019^). The infiltrated inflammatory monocytes in our study obtained CX3CR1 during transformation into macrophages. However, it was unclear whether the transformed cells had the same roles as resident macrophages. Further studies using single-cell RNA sequencing with comprehensive macrophage-specific markers are required to clarify these issues.

This study had some limitations. First, we lacked functional results. In our previous study, depletion of monocytes using *in vivo* blockade of CD11b did not prevent noise-induced hearing loss, similar to the findings of the current study using clodronate liposomes (data not shown) (Bae et al., 2021c). Another limitation of this study was the relatively short interval evaluated after acoustic overstimulation. Indeed, Zhang et al. reported monocytes were found in the cochlea at 20 dpn which is a far longer interval compared to ours (Zhang et al., 2020). The short interval was used because we aimed to focus on the infiltration and transformation of inflammatory monocytes, and this process was found to be complete within only 5 dpn. Furthermore, as Zhang et al. reported in 2021, neutrophils infiltration after acoustic overstimulation is correlated with the expression of CX3CR1 protein (Zhang et al., 2021). Given their results, wild-type mice may show much fewer neutrophils after acoustic overstimulation. The quantitative analysis (e.g., flow cytometry) using wild type mice should be performed in the future study. Nevertheless, it is definitive that inflammatory monocytes are the major population rather than neutrophils after acoustic overstimulation and there is no massive influx of neutrophils. This is a unique inflammatory process compared to bacterial infection and is contrary to the result of Rai et al reported. (Rai et al., 2020; Bae et al., 2021b). Our findings are not limited to confirming the time-course of these biological processes. Indeed, the results of this study may also provide insights into the mechanisms regulating the innate immune response in the cochlea. In different target processes (i.e., cytokine expression, immune cell infiltration, and macrophage transformation), the treatment schedule should be carefully selected based on the appropriate time course of several days.

In conclusion, our findings showed that inflammatory monocytes from the bloodstream infiltrated the inferior part of the spiral ligament within 1–2 days after acoustic overstimulation. Neutrophils were not a major type of phagocyte in the cochlea during this process. Infiltrated monocytes then transformed into macrophages by upregulating CX3CR1 and downregulating Ly6C within 5 days after acoustic overstimulation. Given these results, treatment to control immune response after a noise-induced hearing loss should be applied as soon as possible and sophisticatedly scheduled according to the target process to regulate.

## Data Availability Statement

The datasets presented in this study can be found in online repositories. The names of the repository/repositories and accession number(s) can be found below: https://www.ncbi.nlm.nih.gov/bioproject/?term=PRJNA800193.

## Ethics Statement

The animal study was reviewed and approved by Yonsei University Health System, Institutional Animal Care and Use Committee (approval number 2019-0182).

## Author Contributions

SB designed the experiments. SS and HP performed the experiments. SB and JJ wrote the manuscript, analyzed the data, and reviewed the manuscript. JC and NS supervised the study design. All authors contributed to the article and approved the submitted version.

## Conflict of Interest

The authors declare that the research was conducted in the absence of any commercial or financial relationships that could be construed as a potential conflict of interest.

## Publisher’s Note

All claims expressed in this article are solely those of the authors and do not necessarily represent those of their affiliated organizations, or those of the publisher, the editors and the reviewers. Any product that may be evaluated in this article, or claim that may be made by its manufacturer, is not guaranteed or endorsed by the publisher.

## Footnotes

1
https://biit.cs.ut.ee/gprofiler

2
http://www.kegg.jp/kegg/pathway.html

## Abbreviations

Dpn: days postnoise
CX3CR1: fractalkine receptor C-X 3-C motif chemokine receptor 1
ABR: auditory brainstem response
IP: intraperitoneal injection
APC: allophycocyanin
3D: three-dimensional
PBS: phosphate-buffered saline
PE: phycoerythrin
DEGs: differentially expressed genes
GO: Gene Ontology
KEGG: Kyoto Encyclopedia of Genes and Genomes
RT-qPCR: Reverse transcription quantitative real-time polymerase chain reaction
Il1- β: interleukin 1 beta
Il-6: interleukin-6
Ccl2: C-C motif chemokine ligand 2
ANOVA: analysis of variance
ICAM-1: intercellular adhesion molecule-1.
