# Supplementary material for: The Time Course of Monocytes Infiltration After Acoustic Overstimulation
Source: Front Cell Neurosci. 2022 Apr 12;16:844480. doi: 10.3389/fncel.2022.844480 (PMC9039292; doi:10.3389/fncel.2022.844480)
Supplement: Supplementary file 1 [file Data_Sheet_1.pdf]

Supplementary Table 1. Cell counts in flow cytometry of the cochlea. Data are shown as mean ± SEM.

**Cell types**	**Control**	**1 dpn**	**2 dpn**	**3 dpn**	**5 dpn**
Lymph + Live + CD11b (Myeloid cells)	62.5 ± 12.3	198.3 ± 51.6	304.3 ± 46.7	288.8 ± 48.24	288.3 ± 68.58
Lymph + Live + CD11b + $Ly6G^-$ + CX3CR1 + F4/80 (Monocytes/Macrophages)	44.5 ± 13.4	146.3 ± 40.0	235.3 ± 41.0	211.8 ± 40.0	203.5 ± 52.6
Lymph + Live + CD11b + $Ly6G^-$ + CX3CR1 + F4/80 + $Ly6C^-$ (Macrophages)	42.8 ± 13.7	57.8 ± 19.2	86.0 ± 9.9	135.8 ± 32.1	179.5 ± 47.1
Lymph + Live + CD11b + $Ly6G^-$ + CX3CR1 + F4/80 + $Ly6C^+$ (Intermediate Monocytes)	0.3 ± 0.3	16.5 ± 7.4	55.0 ± 9.1	50.3 ± 10.1	21.8 ± 5.5
Lymph + Live + CD11b + $Ly6G^-$ + CX3CR1 + F4/80 + $Ly6C^{++}$ (Inflammatory Monocytes)	1.3 ± 0.8	72.0 ± 28.2	94.3 ± 25.4	25.3 ± 11.7	1.3 ± 0.5
Lymph + Live + CD11b + Ly6G (Neutrophils)	2.0 ± 1.4	8.0 ± 3.9	6.3 ± 2.7	1.3 ± 0.3	2.5 ± 1.6

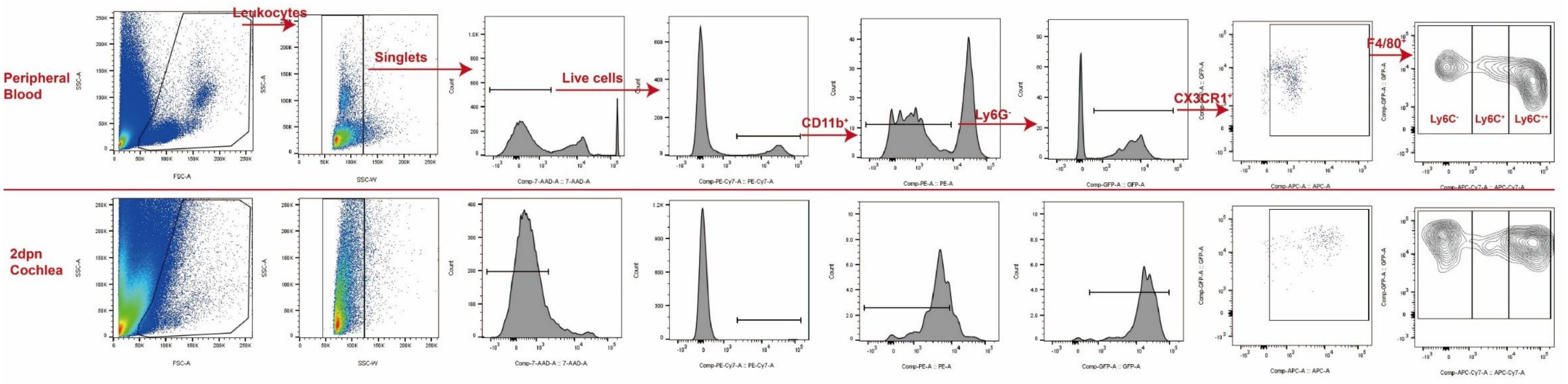

Supplementary Figure 1. Gating strategy for inflammatory monocytes in the cochlea using peripheral blood as a reference. *SSC-A*: amplitude of side scatter, *FSC-A*: amplitude of forward scatter, *SSC-W*: width of side scatter, *7-AAD*: 7-aminoactinomycin D, *PE-Cy7*: CD11b, *PE*: Ly6G, *GFP*: CX3CR1, *APC*: F4/80, *APC-Cy7*: Ly6C, *2dpn*: 2 days postnoise.

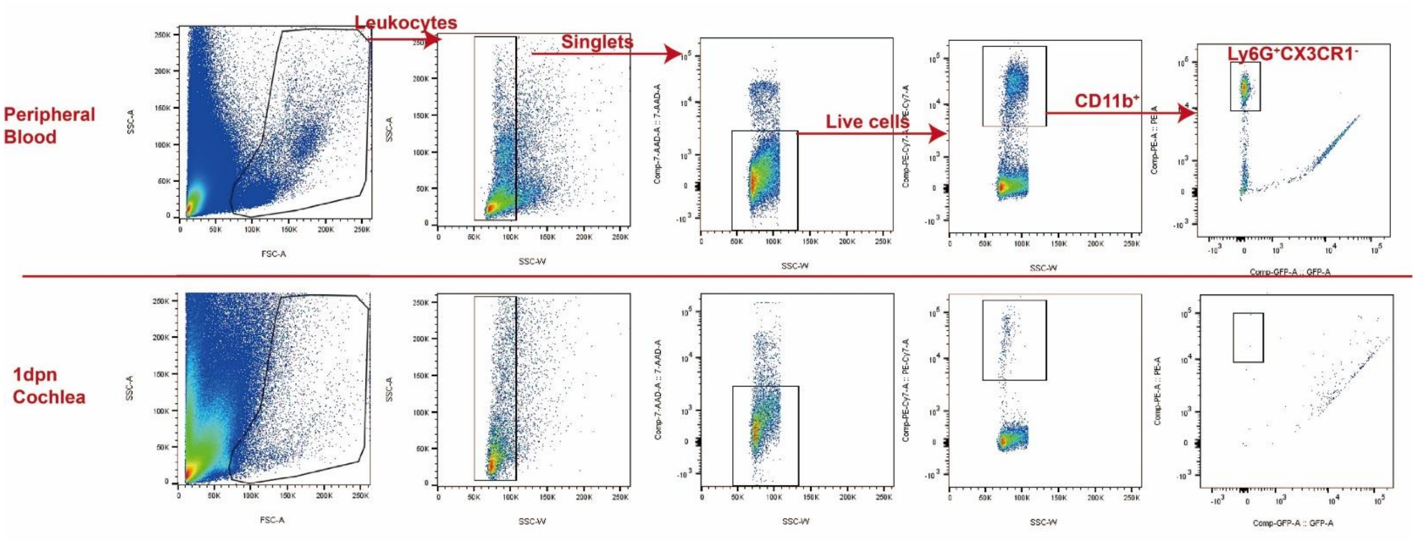

Supplementary Figure 2. Gating strategy for neutrophils in the cochlea using peripheral blood as a reference. *SSC-A*: amplitude of side scatter, *FSC-A*: amplitude of forward scatter, *SSC-W*: width of side scatter, *7-AAD*: 7-aminoactinomycin D, *PE-Cy7*: CD11b, *PE*: Ly6G, *GFP*: CX3CR1, 1*dpn*: 1 days postnoise.

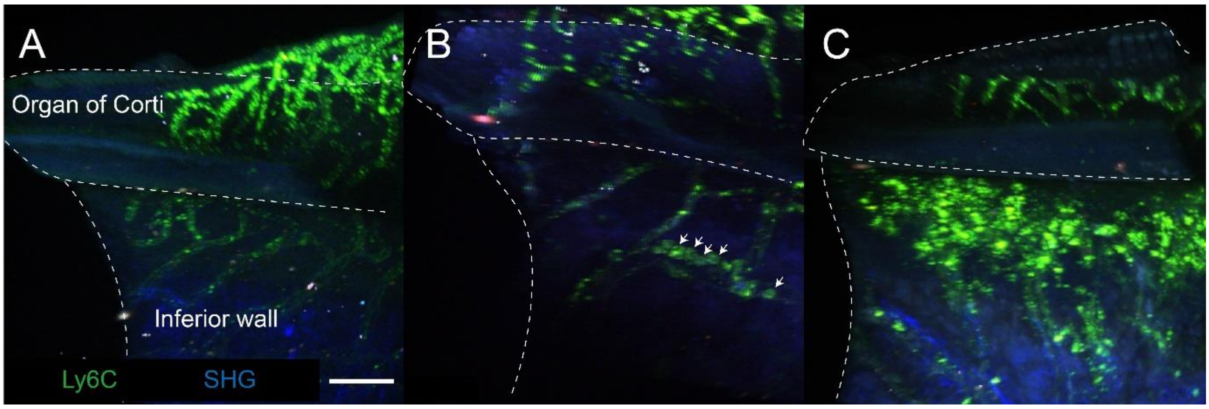

Supplementary Figure 3. The lower portion of the lateral wall of the cochlea was visualized (3D) using two-photon microscopy. (A) Control cochlea. (B) Cochlea at 1 day after acoustic overstimulation. (C) Cochlea at 2 days after acoustic overstimulation. Arrows indicates aggregated monocytes. *Green*: Ly6C, *Blue*: second harmonic generation (SHG) of collagen fibers in the bone. Scale bar = 50 μm. Note that Ly6C also stained the capillary vessel wall.

Supplementary Video 1. Monocytes crawling inside the collecting venule were observed by two-photon live imaging. Immobile large cells at the edge of vessel are resident perivascular macrophages. Acquisition rate was 1 frame per minute. Images were stacked at 30 μm. *Green*: Cx3cr1-GFP, *Red*: Texas-red dextran, *Blue*: second harmonic generation of bone.
